# Dying, death and bereavement: a qualitative study of the views of carers of people with heart failure in the UK

**DOI:** 10.1186/1472-684X-8-6

**Published:** 2009-06-16

**Authors:** Neil Small, Sarah Barnes, Merryn Gott, Sheila Payne, Chris Parker, David Seamark, Salah Gariballa

**Affiliations:** 1 School of Health Studies, University of Bradford, 25 Trinity Road, Bradford, BD5 0BB, UK; 2 Section of Public Health, School of Health and Related Research, University of Sheffield, Regent Court, Regent Street, Sheffield, S1 4DA, UK; 3 Sheffield Institute for Studies on Ageing, University of Sheffield, Elmfield, Northumberland Road, Sheffield, S10 2TU, UK; 4 International Observatory on End of Life Care, Division of Health Research, University of Lancaster, Bowland Tower East, Lancaster, LA1 4YT, UK; 5 Nottinghamshire County Teaching PCT, Birch House, Mansfield NG21 0HJ, UK; 6 The Peninsula Medical School, University of Exeter, The John Bull Building, Tamar Science Park, Research Way, Plymouth, PL6 8BU, UK; 7 Faculty of Medicine and Health Sciences, United Arab Emirates University PO Box 17666, Al-Ain, UAE Faculty of Medicine, UAE University, Al Ain, United Arab Emirates

## Abstract

**Background:**

This paper explores carers' views of dying, death and bereavement for family members who had recently died with heart failure adding to a growing literature on end of life experiences for people with conditions other than cancer.

**Methods:**

Twenty interviews were conducted with bereaved carers of older people with heart failure (HF) who had been participating in a longitudinal study. Carers were approached in writing 3 months after the death. Interviews were transcribed verbatim and analysed thematically with the assistance of NUD*IST.

**Results:**

Findings were grouped into three time periods: prior to death; the death itself and bereavement. Most carers found discussions about end of life with their family member prior to death difficult. Dissatisfaction with the manner of the death was focused around hospital care, particularly what they believed to be futile treatments. In contrast deaths in the home were considered 'good'. Carers adopted a range of coping strategies to deal with grief including 'using their faith' and 'busying themselves' with practicalities. There was some satisfaction with services accessed during the bereavement period although only a small number had taken up counselling.

**Discussion:**

Our findings suggest that an absence of discussion about end of life care wishes with family members or health professionals is a barrier to advance care planning. Carers' perceptions about prioritising making the dying person comfortable can be in conflict with doctors' decisions to treat. Whilst carers report a range of strategies adopted in response to bereavement there is a need for continued support for vulnerable carers after the death of the person with HF.

## Background

Heart failure, predominantly a disease of older people, affects approximately 7% of those aged 75–84 and 15% of those aged 85 and older in the UK [1]. The primary goal of heart failure management is to maximise life expectancy and improve quality of life [2], where prognosis is generally worse than that in either breast or prostate cancer [3]. People with heart failure are, in the main, older and more likely to experience an unpredictable disease progression than people with cancer [4-6]. Service models and conceptual frameworks for people for whom the focus of care is on improving quality of life in the context of life-limiting conditions have been shaped around cancer experiences. More recently there has been some increase in attention paid to providing examples of palliative care services for non-cancer care and in particular for heart failure [7-9] and an increase in the extent to which heart failure is considered in palliative care research [10-13]. Also a series of policy documents, including NICE guidance on heart failure [2], a National Service Framework on Coronary Heart Disease [14] and, more generically, the End of Life Care Strategy [15] have included this group of patients.

However there is only limited knowledge about how patients with heart failure regard death and dying [16] and there is evidence of an absence of discussion about choices of care modalities or even the implications of diagnosis between clinicians, patients and families [10]. Attempts to change practice to respond to such shortcomings have encountered barriers to discussion inherent in the characteristics of the disease, as well as in the reported stances of both patients and heart failure specialists [17].

A key area in end of life care in heart failure, as in other areas of palliative care, is the experience of carers. Whilst the rhetoric of specialist palliative care places high priority upon addressing the needs of carers, in practice research exploring carer experience at the end of life is limited to some key areas and there are notable gaps [18]. Where substantial amounts of research on caring do exist, in social gerontology [19], this work is rarely referred to in end of life care. Specifically, while there is now a considerable amount of research considering carers' needs and the adverse effects of caregiving in palliative care some groups remain under-researched, including people with cardiovascular disease and heart failure [20] where there is only limited work on informal care [21].

This paper seeks to add to the knowledge we have concerning end of life care for people with heart failure. It assesses carers' views on end of life care, the circumstances of the death and bereavement experiences. This allows us to reflect on the applicability of models of the good death in palliative care that have emerged, in the main, from cancer experiences in the context of the specific circumstances of heart failure. Those circumstances, introduced above, also prompt an engagement with debates about what constitute futile interventions in end of life care for people with heart failure.

## Methods

This study, conducted between 2003 and 2006, was approved by a Multi-centre Ethics Committee (Cardiff), with research governance approval obtained from relevant Primary Care Trusts.

The qualitative methodology used semi-structured interviews undertaken in the carers' home or by telephone. Participants were the bereaved carers of patients taking part in a larger quantitative survey exploring palliative care services for 542 heart failure patients over a two year period. More detailed information about the methods employed in the larger study and strategies to involve ill older people in research can be found in another publication[22]. To summarize, 542 people aged >60 years were recruited from 16 GP surgeries in four areas of the UK: Bradford, Barnsley, East Devon and West Hampshire. Patients were considered eligible for recruitment into the study if they were >60 years, could speak English, did not have evidence of significant cognitive impairment, and had self-reported New York Heart Association Classification (NYHA) class II-IV. (This classification indicates that they had a burden of disease that varied from slight limitation of mobility through marked limitation when they would be comfortable only at rest to, in class IV, a situation where a patient has to be at complete rest [23].) All the family carers who had consented to be approached, and who had been bereaved during the study, were contacted in writing 3 months after the death of the study participant by the researcher managing the project (SB) and invited to participate in an interview. Of the 44 possible participants twenty consented and were interviewed.

### Data collection and analysis

The interview covered the issues of satisfaction with care, circumstances of the death, support provided after the death and thoughts about how care for people with heart conditions could be improved. To allow for any further topics which may not have been discussed the interviewer asked whether the participant would like to share anything further about their experiences.

Interviews were audio-taped, with participants consent, and transcribed verbatim. The transcriptions were first checked by the interviewer (SB) and any identifying details were removed. The qualitative software computer package NUD*IST (QSR International Pty, Doncaster, Victoria, Australia) was used to store and retrieve sections of the data. Two researchers (SB and MG) read all transcripts to ensure familiarity with the data and then one transcript was coded separately by each of them. The final coding from each researcher was compared and an agreed coding frame was constructed, discrepancies were resolved by consensus. Data were coded and analysed to identify common descriptive themes, which were grouped into clusters. Data collection and analysis were conducted concurrently. Coding was grounded in the data rather than decided *a priori*. Following basic-level coding broader themes were identified and the interrelationship between them was explored and agreed. Divergent cases within each theme were identified and discussed.

## Results

Of the 44 bereaved family carers approached by letter to take part in an interview, 20 (46%) agreed, 12 (27%) gave a negative response and 12 (27%) did not respond at all. The 20 bereaved family carers interviewed came from all four study areas: 6 were from East Devon; 5 from West Hampshire; 4 from Barnsley and 5 from Bradford. Seventeen of the 20 were female. Thirteen were either a spouse or partner of the person with heart failure, 6 were daughters and one was a son. Seven carers were under 60 years of age, 5 were between 60 and 70 and the remaining 8 carers were over 70. Six of the interviews were conducted in the participants' home or place of their choice and 14 interviews were conducted over the telephone. All of the interviews were audio taped and lasted between 20 and 60 minutes.

The findings fell into three time periods; the period prior to death, the death itself and the bereavement period. Most family carers found discussions about what might happen and what the patients' preferences were as death approached difficult. There was little discussion reported with health professionals in the period leading up to the death. Dissatisfaction with the circumstances of the death was focused around hospital care and specifically about interventions that were perceived as unnecessary. Planned deaths in the home were considered to be 'good deaths'. Those who discussed what might happen after death did so in the context of their religious beliefs. Many of the family carers were happy with the professional support they had received during their bereavement, a small number had taken up bereavement support. The main source of support during this period was from friends and family members. Some of the carers described how grief had affected them, the coping strategies they used and how, in some cases, their bereavement had been followed by depression.

In presenting the results in some detail we have divided our interview material into these three time periods and then subdivided into the clusters of descriptive themes identified in our analysis.

### The period prior to death

Our interviews included examples of how dying and death were discussed and how its discussion was avoided. Most discussions were between carers and patients, few with health professionals were reported.

#### Preferred ways to die

A small number of people had discussed with their family carer that they would prefer to die rather than survive a crisis and be helpless or immobile. Sudden death is compared favourably with a death that is accompanied by pain or dependency.

"He basically didn't want to be bed ridden. He had said that he would rather ... I won't say die a fit man but he didn't think it would be any life ... he wouldn't want to live if he was bed ridden." *[daughter, aged 59]*

"... we had a friend of ours that died just like that, you know, he used to say, "I want to go like that" he said, "I don't want to go through pain"." *[wife, aged 72]*

Although several of the carers had discussed dying with their family member, a number reported that this was a difficult area for them. Difficulties were most often reported when the family carer was the son or daughter of the patient participant. In some cases discussion was delayed until very late in the illness.

"...it was only in the week before he died that he began to talk about if this happens or if that happens and I don't want all that extra stuff." *[son, aged 47]*

In one case, the patient wanted to discuss end of life issues, but his daughter found this difficult. She felt that this was a weakness on her part and resolved that she would discuss it if her father broached the subject, although when he did, albeit a little indirectly, she was still not able to respond.

"I'm not very good on death to be honest with you and when he would say to me things like, "oh sometimes I don't feel I want to be here" and I'd say "oh don't be silly dad you've got years left" and all this sort of thing ... some people can talk about these things but I'm not very good at it. I don't like the idea of death from a personal point of view and I'm very frightened about it ..." *[daughter, aged 54]*

#### Reconciled to death

In a small number of cases discussions were reported that looked beyond the process of dying and the event of death. Carers typically used a religious language, for example they spoke of the expectation that death is followed by joining other deceased family members.

"... he really wanted to be with my mother and he had the faith to feel that when he died he would be back with her ... if you've got the faith where you feel you are going to be with them then perhaps death doesn't seem such a bad option." *[daughter, aged 54]*

Discussion of death was facilitated in one case by both the patient and family carer having a sense that their faith both reconciled them to death and gave a structure to the planning of its practical consequences. When asked if he had discussed death with his wife, this family carer responded:

"absolutely, and the basis of that was, and always has been, our faith and religion. We're Church of England and we both agreed a long time ago that we would be buried together and she's buried 500 hundred yards up the road from me here, we live right next door to our church in this little village, and we discussed everything and she was a very strong person, ....and nothing was left unsaid or undone. So everything was planned and we were always conscious of the fact that we are mortal creatures and that one or the other of us is going to die before the other ..." *[husband, aged 62]*

### The death itself

The death as an event was discussed in two main ways, the actual circumstances of it and in particular its suddenness, and the care that was available when it occurred. Accounts of care were characterised by the different way hospital and home death were experienced.

#### Sudden death

Several of the carers reported that their family member had experienced a sudden death or died in their sleep. This type of death was generally regarded as being peaceful.

"I keep looking at it that the end when it came was very peaceful and that to me was the main thing. I didn't want him suffering and ... I'm glad in a way ... not glad he's dead but glad in a way that it was as peaceful as it was. I mean none of us want to die but when we go that way is one of the best isn't it, just to go to sleep ..." *[daughter, aged 57]*

One person had cancer as well as heart failure, the family carer hoped their husband would die from heart failure.

"We always thought cancer would have been it you know, but ... the way he went, I presume he wasn't in any pain. I just think his heart just went. I think if it had been cancer it would have been a bit more dramatic, I would have thought because it's very painful isn't it? I should imagine it would have been a very long drawn out death and I don't think ... I always said I'd rather him ... if he ever went I'd rather it be his heart rather than his cancer."

[wife, aged 72]

#### Sudden death can create specific practical problems

"The most stressful thing was actually when he died because they class it as a sudden death and we had to have the police and everything and that wasn't pleasant ... they basically had to guard the body until a doctor came and certified the cause of death." *[daughter, aged 59]*

#### Hospital or home death

The main area of complaint by family carers about the actual death of their relative, concerned care received in hospital. Several carers felt that there were too many unnecessary interventions and in some cases they perceived staff to be unsympathetic.

"She (registrar) decided he needed a stomach x-ray because his stomach was very bloated but he couldn't eat or drink so she ordered this x-ray to be done. So less then an hour before he died, poor man, there were three of us trying to lift him onto an x-ray plate because obviously he had to go underneath. He was talking to me and I knew that he knew that he was dying." *[wife, aged 47]*

"Then he just got weaker and weaker, he literally could move nothing but his head. He had this dreadful, dreadful registrar who came in who decided that she must find out what was wrong and she kept saying, 'well just tell me what's wrong with you, you just keep going on about these symptoms, they're not specific enough', and she was really arsy and very unpleasant and I could have thumped her." *[wife, aged 47]*

One family carer described his wife as having 'died' at home. She was resuscitated, and kept alive on life support for six weeks in hospital. When asked about her date and place of death, her husband considered this to be at home before resuscitation,

"Her eyes were wide open and she wasn't breathing and her mouth was open and I knew that we were in trouble with this one. I don't know how long she had been like that, it wouldn't have been many seconds, but that is actually when she died. I screamed at God not to let her die and he didn't, he brought her back to life again in my arms and, .. she was kept alive in hospital for another six weeks ... and she lived, albeit artificially, because she was on total life support in hospital where they tried to resuscitate and revive her, and whilst she gained some degree of consciousness it was very, very little ..." *[husband, aged 62]*

When offered the option, some participants preferred less invasive treatments and palliation. For example, one family carer reported that her father was given a choice in the hospital of either having an invasive operation or being 'made comfortable' and left to die.

"They said there were two options: they would operate with very little chance of him coming round. If he did come round he would be in intensive care for a very long time. If he survived intensive care he would probably always have to be in some kind of hospital type situation ... or they could just make him comfortable, pain free, and let him just leave us that way, and ... we were all involved in the choice but ... he chose that was the way to go ... He was very conscious of us being there, we didn't know quite how it was going to happen, but he was also very conscious of my mother being there, so for him certainly I'm sure it was the best option." *[daughter, aged 54]*

This option of being 'made comfortable' was supported by the wife of another participant who stated that she could not see any advantage to resuscitating her husband, if his quality of life would remain poor.

"I'm a great believer in what's the point of keeping somebody alive just for the sake of keeping them alive when you know that the quality of life is going to be appalling and you know the end is not far away anyway." *[wife, aged 47]*

Although some family carers reported particularly distressing deaths in hospital, another carer viewed her husband's hospital death as positive. She felt he was getting the treatment he needed, which she did not feel he would receive at home.

"...he saw absolutely everybody I should think in [hospital] and they ... were very good and very informative and we were able to be with him the whole time even when he was having his examinations." *[daughter, aged 54]*

While death at home was generally seen as a "good death" one carer was pleased her mother died in a nursing home. She felt that dying at home would have left her with unhappy memories.

".... there isn't a corner in my house that's gloomy now because she wasn't here when she went." *[daughter, aged 69]*

The small number of family carers whose spouses had died at home viewed this as positive and considered it a 'good death'. They felt that the end of life care delivered by nurses at home had facilitated this and they welcomed the support they had been given in the last few days before the death.

"... she [nurse] took his pulse and she said, "he's on his way out", and she said, "both hold on to him" and my sister-in-law sat one side and I got on the bed and we held on to him and he died. She kept feeling his pulse and he had gone ... it was marvellous and he had a very peaceful death. ... I'm so glad I kept him at home. It was wonderful really." *[wife, aged 76]*

In a small number of cases, family carers recalled usual medication being stopped prior to death. This was generally thought to be positive as it was apparent in these cases that any further intervention would not be effective or appropriate.

"... and the funny thing was when the doctor decided that, you know, I was going to keep him at home, he stopped all his tablets, and he was taking an enormous amount of tablets every day, it used to take me about half an hour to sort them out in the morning, and he stopped the lot." *[wife, aged 76]*

### The bereavement

#### A gap in their lives

As expected, all of the carers experienced grief after the death of their relative.

"I just find that at times ... I still think I should ring him because we used to ring so regularly and you'd find that you'd wake up on a Saturday, "I think I should go down there", because that's what you did. So those are the things I find most ... well those are the things that keep popping up." *[son, aged 47]*

For this bereaved spouse, her own disabilities became more apparent after the death of her husband, affecting her ability to socialise easily with other people.

"... I have one problem, I'm deaf, not too badly deaf but I am, but it didn't matter to me with [husband] because he would shout and I could hear him. I do have a job to hear other people, you know, so I find that a problem." *[wife, aged 76]*

Some of the carers were deeply distressed. Both the duration and intensity of their grief surprised them.

"... it sort of comes over you and the grief hits you and it really is like falling into a deep pit, it's a horrible feeling and I couldn't sort of get out of it, and I couldn't see any way out ... because I'm seventy five, I shall soon be seventy six and I couldn't see what I could do with my life at that age, and I'd been with [husband] for fifty one years." *[wife, aged 75]*

"... I'm actually taking it very badly and the doctor said ... I've never been depressed but he says I've got all the signs of depression ... I mean I'm worse now than I was at the beginning because I had ... well I still have a lot to do but ... I mean I keep the place and I keep the garden going and I do all the things, but there's no interest like there was." *[wife, aged 75]*

#### Strategies

The bereaved carers described a variety of strategies they used to deal with the grief.

"Time as they say is a great healer, but nothing really ... you just have to learn to work through grief because you can't go round it, you can't cut it out, you can't put it to the back of your mind because some time or other you have to deal with it and you have to face it; the permanency of it. To realise that the person that meant the most in your life has gone for good – on this earth." *[husband, aged 62]*

Some of the carers tried to think about positive things relating to the death of their spouse to help them during the bereavement period.

"I try to think that he had his brain to the end. Okay he couldn't do what he used to do or anything like that but he could do a few things ... I must hang on to that because he would have hated not to do for himself." *[wife, aged 75]*

"Just getting on with it" was a strategy mentioned by several of the family carers, particularly the spouses.

"It's just upsetting when they go and you just get on with it don't you, there's not a lot you can do. Get used to the bed on your own and them not being there. It's just what thousands of other folk have to put up with as well isn't it? I keep his pictures there and I keep looking at them." *[wife, aged 70]*

Several carers discussed busying themselves with practicalities of probate and funeral arrangements.

"I decided to take on the probate and all that sort of thing so I've been dealing with all that. I'm not particularly that way inclined but it's probably a good thing in a way because it does focus your mind onto something else." *[daughter, aged 54]*

A small number of bereaved wives reported becoming more active since the death of their spouse.

"... I'm just starting now to get myself organised again. I've somebody coming this week about me sitting with elderly people just for companionship you know, just visiting, because I said to her, "well I'm seventy myself" but I don't feel old and I've always had a soft spot for old people anyway." *[wife, aged 71]*

Support.

Bereaved carers discussed support needs both in terms of what they felt they needed or wanted from the NHS and what they sought from families, friends and neighbours. A number of carers were happy with the support they had been offered. Three family carers had taken up the offer of bereavement counselling.

"There's a fairly well-established bereavement service which is quite well managed in terms of the death certification process and you know, it's handled by people who've got bereavement counselling experience there ... The actual written information you know, was reasonable. I mean, it felt genuine. The material that comes in the bereavement pack with the certificate is actually extremely helpful at the time when you need some." *[son, aged 47]*

However most reported receiving no bereavement support, although there was some sense that professional help might have been welcomed. At the very least this participant would have like to receive a letter of condolence from their GP.

"... I never heard another word from the practice at all ... I got nothing from his GP. Not even a phone call or anything." *[daughter, aged 54]*

Social support could also be difficult to access. A small number of bereaved carers felt that other people were often uncomfortable talking about the death of their relative, even when encouraged to do so.

"... I tell myself I'm quite strong and I am because I try not to get upset because it embarrasses people, they ask how you are and they want to know, but they don't know what to do if you get all upset and that, you know, and yet on the other hand if you're putting on a brave face they think, "she's not bothered" you know, there's no happy medium is there really?" *[wife, aged 71]*

## Discussion

Consistent with other studies [10] we have highlighted a reluctance to discuss end of life wishes between family carers and people with heart failure [24]. However carers did have ideas about the sorts of death that they preferred and they believed the person they were caring for preferred. These were sudden death and death at home. We see here the beginnings of a construct of a good death that might accompany heart failure. It differs from constructs of the good death developed in palliative care which characteristically include open awareness and a gradual progression towards death [25] although it shares a preference for death at home. It is worthy of note that any constructs of the good death have to be tentative given a considerable variety of reported experiences, in Low and Payne [26] cancer patients in a hospice showed a preference for a sudden death when asked to define a 'good death'.

A further area of concern expressed in the interviews we report links personal experiences with care planning. There is a rejection by carers of what are seen as futile interventions at the end of life. The stories of distress created by these can, at the very least, be seen as calls for better training and supervision for medical staff [27]. They may also be illustrative of a wider difficulty in arriving at a shared understanding between clinician, patient and patient's families about what a futile intervention is [28].

In the discourse of medicine futility is a term used to define situations in which treatment provides no, or little, chance of survival with a meaningful quality of life [29]. A one per cent chance of survival has been suggested as a cut off point [30]. Swanson and McCrary [31] found that different risk calculations were adopted for people who have a progressive and incurable disease, futility was assumed even if the percentage chance of survival was greater than one per cent. But as we have seen, even with a less restrictive definition of futility, there are still differences between medical staff and family carers. Family carers approach to deciding what was a desired intervention and what was futile, was closer to a dictionary definition of futility as not just something ineffective or useless but something lacking weight or importance – something frivolous, lacking purpose. There is even a definition of futility that sees it as loquacity, an inability to hold one's tongue (Shorter Oxford English Dictionary). Adopting this definition means learning more about symptoms by taking X-rays, or making repeated requests to have symptoms described, is futile. Sustaining life by technology is futile because, for the carer, the person has already "died". Indeed all our examples, X rays, questioning, use of life support, are examples of medical loquacity. They embody an imperative to "do to" in contrast to the carers wish to "be with" [32]. What to "be with" means is vividly encapsulated in the carer in this study who spoke of stopping tablets, here the doctor understood that this was a way to be with his patient and their carers, or by the nurse, sister and wife holding the person as they died. One of our carers did like the idea of end of life care in hospital because of the presence of all the technology that might be needed, but it was the sense of reassurance in the potential to respond that was valued, not the use of the technology.

Ågård et al's study [33] illuminates many of the points about futility raised above. This study examined attitudes to cardiopulmonary resuscitation (CPR) in patients with heart failure. Even with an observed in-hospital cardiac arrest, survival rates to discharge after CPR for patients with heart failure is very poor, indeed moderate-to-severe heart failure is one of the strongest independent predictors of in-hospital mortality after CPR. Even if the patient survives there are many examples of negative effects associated with CPR, neurological and functional impairments most notably. Ågård and colleagues identify that those patients with heart failure who are asked to express a view about CPR are asked relatively early in their illness. At this point they do not consider their burden of symptoms to be great and they answer that they would want CPR. If they were asked when they have more severe symptoms, say classes IIIb-IV (NYHA) then the answer may be closer to that provided by their carers in the study this paper is reporting, that is they may prioritise comfort over prolonging life. Seeking meaningful consent via patient views then becomes something that should be understood as a process and not an event – a person is likely to change their view as the impact of the progressive disease becomes more pronounced [34].

We can then refine our emerging model of a good death. So far we have identified sudden death and death at home as two key components. Having considered the experience of what is seen as futility we can add support for an approach of seeking comfort and avoiding what carers see as unnecessary interventions. Many of our carers have been caring for their family member over a long period and through increasing debilitation and periods of crisis. They recognise that death can be timely even if this recognition has not always been openly acknowledged either by themselves or by the person who is dying.

We now take this emerging conceptualisation of aspects of the good death in heart failure and add the additional concerns of, first, patient choice and then carer needs. In this contested context of different perspectives held by professionals and by family carers a route forward that is consistent with prevailing policy guidelines, like the End of Life Care Strategy [15], is to ensure where possible that it is the patient who decides about end of life care (not the clinical team or relatives). We have described one example where this happened, the hospital giving the patient the choice of an operation or being made comfortable. This was done at a time where his family were with him and were able to honour and support his decision to choose comfort. Our findings have shown that the sorts of advance discussions and directions that are required to ensure patient choice are not often present. Interrelated features of uncertainty of patterns of progression and a reluctance to acknowledge the terminal nature of progressive heart failure [16] by all parties involved in care continue to inhibit such discussions. Even if discussion well in advance did happen it would not be enough. Murray et al [7] present an argument consistent with ours in arguing that plans for what the patient considers a good death ought to begin early but be reviewed subsequently as their condition changes, and heart failure is a condition that can change often.

But there is another component of the good death that needs to be put in place in heart failure care. Our interviews have presented examples of the need to see the patient in a social context, and to recognise that the death of the patient does not stop the need to plan care.

Our interviews highlight that some carers are very aware of needs of their own after the death of the person they are caring for. While this is likely to be true of all carers, and while many of the needs are about finding new interests or new sources of social support, in a small number of cases there is a need for a professional input that may reduce future demands generated by socially isolated and depressed former carers [35]. The interviews also suggest that a positive experience of the death helps in the process of adjustment. The sense that the person died in a peaceful way that felt like "the best option", or that the ease of the death was "wonderful" resonates with the quote from Dame Cicely Saunders on the front cover of the Government's End of Life Care Strategy that says, "How people die remains in the memory of those who live on" [15].

This is a small study and significant numbers of those approached to take part either did not respond or declined to be interviewed. It may be that their views and experiences differed from those who are reported here. Those who did respond were given the choice of being interviewed by telephone or in their own home. Our analysis of the resulting transcripts did not identify any differences between these two approaches to data collection and we have not identified the interview type in our results. The study recruited bereaved carers from different parts of the country. We did not identify any regional patterns in our interviews and so have not specified location in our results. The data capture retrospective assessments of the dying and death of the person being cared for, although for some the bereavement experiences were still current, not retrospective. Our reports are from carers who are reporting as proxies when they describe what they felt the experiences of the person who was dying was, for example in terms of their preference for a sudden death, but in the main what they report are interactions between themselves, the person they are caring for and in a number of examples members of the health services. Reiterating our title we present carers views and in this sense our informants are primary sources of data and not proxies. A review of studies of the validity of proxy responses to capture the experience of end of life care concluded that that proxies can reliably report on the quality of services and on observable symptoms but proxy reports show less agreement with reports from patients on more subjective aspects of the end of life experience, pain, anxiety and depression in particular [36]. We recognise the potential of a longitudinal study in this area that would collect data contemporaneously during end of life care, death and bereavement. We do note however that in the wider study that this paper sits within of 542 people with heart failure only 44 died during the study and that who those 44 were likely to be could not be predicted [4]. We are also aware that we are gathering information from carers who are relatively recently bereaved. The impact of bereavement may shape the way they report end of life care, specifically what carers report during bereavement may diverge from reports they have given during the terminal phase of the patients illness [37,38]. Further, some carers identify feelings of depression and these may impact on the views they express. Research has underlined the impact of depression on quality of life of patients and on the shortcomings of services to both recognise and respond to it [39,40] our study suggests a need to recognise and respond to depression in carers also. Despite its limitations this study has identified conceptual and service issues in relation to the impact on carers of end of life care, death and bereavement in heart failure that both support and add to existing literature.

## Conclusion

Carers of people with heart failure find it difficult to discuss the wishes of their family members prior to death, this is a barrier to advance care planning. They are however clear that they are opposed to futile interventions. They emphasise quality of life rather than an extended life. Death at home is preferred by many but not all family carers. The sense that the death has been a good one helps shape post-bereavement experiences, as does having a religious faith. Bereaved carers are likely to have continuing needs. Some of these are consequent on their age, their own health problems and the length of time they have been offering care and the impact this might have had on their social networks. These needs require a response that combines a focus on supporting and enhancing social networks and, for some, professional input responding to depression.

## Competing interests

The authors declare that they have no competing interests.

## Authors' contributions

NS drafted the final version of the article and was involved in design of the wider study this paper draws on. SB undertook the interviews and developed the initial draft of the article. MG analysed data and designed the wider study this paper draws on. SP analysed data and designed the wider study this paper draws on. CP was involved in design and analysis. DS was involved in subject recruitment, study design and specialist input re heart failure. SG was involved in subject recruitment, study design and specialist input re heart failure and general practice. All authors contributed to the development of the article and all read and approved the final manuscript.

## Pre-publication history

The pre-publication history for this paper can be accessed here:


